# PrEP adopted by the brazilian national health system: What is the size of the demand?

**DOI:** 10.1097/MD.0000000000010602

**Published:** 2018-05-25

**Authors:** Paula M. Luz, Adele Benzaken, Tatianna M. de Alencar, Cristina Pimenta, Valdilea G. Veloso, Beatriz Grinsztejn

**Affiliations:** a Instituto Nacional de Infectologia Evandro Chagas, Fundação Oswaldo Cruz, Rio de Janeiro; b Department of Surveillance, Prevention and Control of STI, HIV/AIDS and Viral Hepatitis, Ministry of Health, Brasília, Brazil.

**Keywords:** Brazil, HIV/AIDS, PrEP

## Abstract

**Background:**

Brazil's response to the HIV epidemic now includes free access to preexposure prophylaxis (PrEP) to populations at substantial risk for HIV infection including men who have sex with men (MSM). We used nationally representative demographic, epidemiologic, and surveillance data to offer estimates for the number of MSM at substantial risk for HIV infection who might be eligible and willing to use PrEP in Brazil.

**Methods:**

Starting from the age/sex-stratified population, we calculated the number of men aged 15 to 64 years, in 5-year age groups, and the proportion of those who report sex with other men during their lifetime. We focused on 11 cities (representing all regions) that are responsible for a significant fraction of the HIV burden of the country and used city-specific HIV prevalence estimates to infer the fraction of MSM who are HIV-negative. We then derived the proportion of HIV-negative MSM under substantial risk for HIV infection defined as having unprotected receptive anal intercourse in the 6 months before study participation. Finally, PrEP uptake among the eligible was inferred from the PrEP Brazil study.

**Results:**

Our results show that PrEP demand in these 11 cities is of 66,120 men aged 15 to 64 years. When we consider the lower and upper bounds for the available parameters, we find that PrEP demand in these 11 cities might vary from 33,378 to 97,962 men. If PrEP is restricted to those aged 15 to 49 years, demand drops by 20%. PrEP demand varies considerably by city, mostly because of the differences in population size and city-specific HIV prevalence.

**Discussion:**

We have shed light on the probable size of PrEP demand in Brazil certain that the incorporation of PrEP as part of Brazil's combination prevention for populations at substantial risk for HIV infection is a necessary challenge. PrEP will not only prevent HIV infections, it will also expand testing among the most vulnerable with the added benefit of offering combination prevention for the uninfected and immediate treatment for those already infected. As such, expected added benefits of PrEP will be earlier linkage to care, prompt treatment initiation leading to health benefits and decreased transmission.

## 1 Introduction

Since its onset, Brazil's HIV epidemic has been concentrated in high-risk populations of large urban centers.^[1]^ Over the decades, Brazil's combination prevention and treatment efforts have included education and prevention campaigns, condom distribution, HIV testing, and antiretroviral treatment coupled with laboratory monitoring. Since 2002, to expand testing, the Department of STI, AIDS, and Viral Hepatitis added the use of rapid tests (finger-prick) in primary care services throughout the country. Nonoccupational post-exposure prophylaxis was included in 2009. Specifically targeting populations at substantial risk for HIV infection, in 2013, 40 nongovernmental organizations were trained in oral fluid rapid testing for HIV to provide access to testing at alternative times and locations. In the same year, antiretroviral treatment was made available to all HIV-infected individuals, irrespective of CD4 cell count. On May 2017, Brazil took 1 step further by announcing free access to preexposure prophylaxis (PrEP) to populations at substantial risk for HIV infection including men who have sex with men (MSM) and transgender women, sex workers, and serodiscordant couples.^[2]^

When examining the latest numbers of Brazil's HIV epidemic, an expansion of the national response is warranted. Although the continued low HIV prevalence in the general population is certainly positive, the epidemic continues to grow and expand in key populations. The number of new infections is rising with a disproportionate impact among young MSM.^[3]^ To help address this growing concentrated epidemic, the inclusion of PrEP follows well in line with other strategies adopted in the past decade.^[4]^ Moreover, PrEP will not only prevent HIV infections, it will also expand testing among the most vulnerable with the added benefit of offering combination prevention for the uninfected and immediate treatment for those already infected. As such, expected added benefits of PrEP will be earlier linkage to care, prompt treatment initiation leading to health benefits and decreased transmission.

The available evidence of PrEP's efficacy and effectiveness in our setting suggests that, similarly to France and the UK,^[5]^ we also need to address the next relevant question: what is the size of PrEP's demand? Accordingly, for the present analysis, we used nationally representative demographic and surveillance data to offer estimates for the number of MSM at substantial risk for HIV infection who might be eligible and willing to use PrEP in Brazil.

## 2 Methods

### 2.1 Demographic data: size of the MSM population

The Brazilian Institute of Geography and Statistics or Instituto Brasileiro de Geografia e Estatística (IBGE) (Portuguese) conducts national census of the Brazilian population and provides inter-census estimates by age and sex for all Brazilian states and the federal district. Starting from the estimated age/sex-stratified 2016 population of each city,^[6]^ we calculated the number of men aged 15 to 64 years, by age group, assuming, for each city, the age distribution as that of the corresponding state. As per estimates provided at IBGE, we considered 5 years age groups: 15 to 19, 20 to 24, 25 to 29, 30 to 34, 35 to 39, 40 to 44, 45 to 49, 50 to 54, 55 to 59, 60 to 64. To infer the proportion of MSM, we used the most recent nation-wide survey of sexual practices and behavior that showed that 3.5% (95% confidence interval [CI] 2.9%–4.3%) of the sexually active men report sex with other men during their lifetime.^[7]^

### 2.2 Surveillance data: size of the HIV-negative MSM population at substantial risk for infection

To infer the number of HIV-negative MSM at substantial risk of HIV infection in Brazil, we focused on 11 cities that are responsible for a significant fraction of the HIV burden of the country while also representing all 5 of its major regions. Ten cities were selected by the Department of Surveillance, Prevention and Control of STI, HIV/AIDS, and Viral Hepatitis of the Ministry of Health for a nation-wide respondent-driven sampling study that estimated HIV prevalence and behavioral practices related to HIV risk.^[8,9]^ The 11^th^ city was São Paulo, the city with the highest number of HIV infections in the country^[3]^ where a time-location sampling study was conducted to estimate HIV prevalence and behavioral practices in the municipality of São Paulo.^[10]^ Both studies assessed adult (≥18 years) men reporting sex with men.

First, we used city-specific estimates for HIV prevalence^[8,10]^ to infer the fraction of MSM that is HIV-negative, which was shown to vary from 76% (95% CI 69%–83%) in Brasília to 95% (95% CI 92%–97%) in Recife. We then derived the proportion of HIV-negative MSM under substantial risk for HIV infection using the percent of men who were sexually active in the 6 months before study participation (varied from 73% [95% CI 66–82] in Santos to 97% [95% CI 96–99%] in Recife),^[8]^ and, of these, the percent of men who reported having unprotected receptive anal intercourse in the 6 months before study participation (36.5%, varied from 24.4% in Itajaí to 40.0% in Curitiba).^[9]^ Accordingly, we defined substantial risk for HIV infection similarly to the epidemiologic sources,^[8,9]^ as having unprotected receptive anal intercourse in the 6 months before study participation. Finally, PrEP uptake among the eligible (60.9%) was inferred from the PrEP Brasil study, a multicentric, open-label demonstration project that assessed PrEP feasibility provided at no cost to MSM and TGW at substantial risk for HIV infection in the context of the Brazilian public health system.^[11]^

## 3 Results

**Figure 1:**
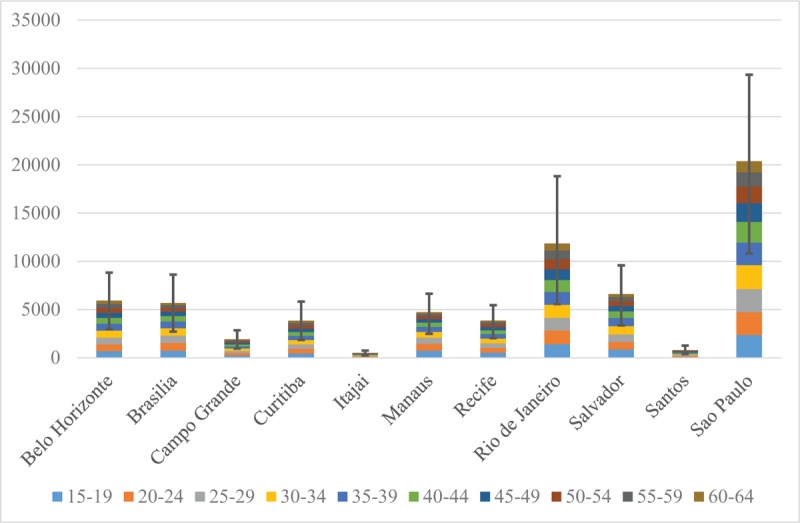
Estimated pre-exposure prophylaxis demand by city and age group.

Our results show that PrEP demand in these 11 cities is of 66,120 men aged 15 to 64 years. When we consider the lower and upper bounds for the available parameters, we find that PrEP demand in these 11 cities might vary from 33,378 to 97,962 men. If PrEP is restricted to those aged 15 to 49, demand drops by 20% to 52,819 men (range 26,670–78,213). Figure 1 shows that PrEP demand varies considerably by city, mostly because of the differences in population size and city-specific HIV prevalence.

## 4 Discussion

We estimated that PrEP demand among eligible MSM aged 15 to 64 years amounts to 66,000 and might reach 98,000 men. Importantly, our estimates are based on one definition of substantial risk for HIV infection, which matches that of the current Brazilian Ministry of Health PrEP Guideline, and other definitions could lead to different estimates. However, other studies have used the same definition yielding upper estimates of ∼50 thousand men who would need PrEP in France and the UK, values not yet adjusted by the proportion of men who would indeed uptake it.^[5]^ In the present, we included the best available estimate of PrEP uptake, although the PrEP Brasil study showed that previous PrEP awareness as well as higher perceived risk of HIV infection increased individuals’ willingness to use PrEP ^[12]^ suggesting that greater awareness of PrEP and of one's own behavior could lead to increased PrEP demand over time. Indeed, given that PrEP literacy is lowest among the most vulnerable,^[12]^ increasing PrEP knowledge is a major challenge to the success of a PrEP program.

An additional challenge will be adherence. Fortunately, week 48 results of PrEP Brasil showed that drug concentrations suggestive of high protection (≥4 doses/week) were achieved by 73% of those retained in the study (83% were retained through 48 weeks).^[13]^ Furthermore, to reach the most vulnerable, health care services where PrEP will be delivered must also provide the necessary accompanying services such as HIV counseling and testing in a stigma-free setting.^[4]^ Indeed, the requirement that it should be in a stigma-free setting cannot be understated.^[14]^ One of the pillars of the Brazilian National Health System is to provide equitable free access to health—a challenge given the country's marked health disparities.^[15]^ High levels of stigma have been shown to correlate with higher odds of unmet prevention needs, with decreased use of testing services.^[16]^ Finally, we stress that future studies should include other populations targeted for PrEP programs which, in Brazil, include transgender population and sex workers, for a more complete assessment of PrEP demand.

In sum, we have shed light on the probable size of PrEP demand in Brazil certain that the incorporation of PrEP as part of Brazil's combination prevention for populations at substantial risk for HIV infection is a necessary challenge.
